# Radiotherapy induced immunogenic cell death by remodeling tumor immune microenvironment

**DOI:** 10.3389/fimmu.2022.1074477

**Published:** 2022-12-01

**Authors:** Songxin Zhu, Yuming Wang, Jun Tang, Min Cao

**Affiliations:** Department of Thoracic Surgery, Renji Hospital, School of Medicine, Shanghai Jiao Tong University, Shanghai, China

**Keywords:** radiotherapy (RT), tumor immune microenvironment (TIME), immunogenic cell death (ICD), immune-checkpoint blockade (ICB), tumor

## Abstract

Emerging evidence indicates that the induction of radiotherapy(RT) on the immunogenic cell death (ICD) is not only dependent on its direct cytotoxic effect, changes in the tumor immune microenvironment also play an important role in it. Tumor immune microenvironment (TIME) refers to the immune microenvironment that tumor cells exist, including tumor cells, inflammatory cells, immune cells, various signaling molecules and extracellular matrix. TIME has a barrier effect on the anti-tumor function of immune cells, which can inhibit all stages of anti-tumor immune response. The remodeling of TIME caused by RT may affect the degree of immunogenicity, and make it change from immunosuppressive phenotype to immunostimulatory phenotype. It is of great significance to reveal the causes of immune escape of tumor cells, especially for the treatment of drug-resistant tumor. In this review, we focus on the effect of RT on the TIME, the mechanism of RT in reversing the TIME to suppress intrinsic immunity, and the sensitization effect of the remodeling of TIME caused by RT on the effectiveness of immunotherapy.

## Introduction

Characteristics of tumor include sustained proliferation, resistance to cell death, angiogenesis, invasion and metastasis, as well as suppression of inflammation and immunity (1). Among them, immunosuppression, an important feature of the tumor immune microenvironment (TIME), is considered to be an important reason of tumor progression and metastasis and has become a therapeutic target for numerous tumor types. Radiotherapy (RT) with highly effective and non-specific in nature is one of the commonly used therapies in the treatment of malignant tumors. RT is regarded as the most effective cytotoxic therapy for treating patients with solid tumors and is used as first-line treatment in approximately 60% of newly diagnosed patients (2, 3). Firstly, RT directly or indirectly induces DNA damage and endoplasmic reticulum (ER) stress, leading to tumor cell death, which is thought to target cancer cells. In addition, non-targeted and systemic effects of RT have also been identified (4). There is growing body of evidence that RT can remodel TIME to alter the original immunosuppressive state, exert anti-tumor effects, and exhibit enhanced immune responses and therapeutic effects when combined with immunotherapy (5–7). This article partly reviewed the impact of TIME on immunosuppression and the effects of RT on TIME, elaborated the mechanisms of reversal of TIME on the suppression of intrinsic immunity, and the sensitizing effect of the remodeling of TIME on the effectiveness of immunotherapy.

## TIME

TIME is the structural and functional niche where tumor cells arise and live, and includes not only tumor cells and extracellular matrix (ECM), but also fibroblasts, epithelial cells (ECs), immune or inflammatory cells, blood and lymphatic vessels, etc (8). TIME, mediated by the secretion of a large variety of factors by a diverse range of cells, forms a local milieu that favors tumor proliferation, infiltration and metastasis. Tumorigenesis is usually accompanied by the activation of innate and adaptive immunity, called functional cancer immunosurveillance, which gradually results in the accumulation of immune or inflammatory cells within the TIME. The immune response plays a dual role in the complex interaction between tumor and host (pro-/anti- tumor) and undergoes cancer immunoediting processes (elimination, equilibrium, and escape), culminating in the formation of an immunosuppressive microenvironment that promotes malignant tumor progression. Natural killer (NK) cells are key cells in innate immunity, relying on granzymes and perforin for direct cell killing without prior sensitization or MHC restriction. In the adaptive immune system, CD4^+^ T cells and dendritic cells (DCs) are important mediators, while CD8^+^ cytotoxic T lymphocytes (CTLs) play the ultimate tumor-killing role. CD4^+^ T cells, mainly T helper cells, broadly play an important adjuvant function in the recognition and clearance of tumor cells, through promoting the proliferation and activation of CTLs, the formation of memory CTLs, and enhancing the antigen presentation of DCs (9, 10). Cytolytic CD4^+^ T cells recognize antigenic peptides presented by MHC-II molecules mainly on antigen-presenting cells (APCs), and are relevant to antitumor immunity in cancer patients (11, 12). DCs are the most important APCs that initiate adaptive immune responses *via* activation of naive T cells (13). DCs cross-present MHC-I molecules to CD8^+^ T cells to induce the production of cytotoxic effector CD8^+^ T cells, known as CTLs (14). CTLs recognize MHC-I molecules expressed by tumor cells and specifically kill tumor cells through Granule exocytosis and Fas ligand (Fas-L)-mediated apoptosis induction (15). Moreover, CTLs release interferon-γ (IFN-γ) and tumor necrosis factor α (TNF-α) to induce cytotoxicity within tumor cells (16). Tumor-associated macrophages (TAMs) are abundant in TME and are major players in the inflammatory response. In addition to promoting tumor cell proliferation and angiogenesis, TAMs suppress adaptive immune responses (17). Cancer Associated Fibroblasts (CAFs), the plentiful stromal cells in the TME, are a major source of extracellular matrix fibrogenic components such as collagen, hyaluronic acid and fibronectin (18). CAFs actively contribute to cancer invasion by modulating distinct malignant processes (angiogenesis, chronic inflammation and ECM remodeling) and therapeutic resistance (19). CAFs control the functional fate of innate and adaptive immune cells in the TIME by secreting cytokines/chemokines and engaging in direct intercellular interactions (20). Moreover, CAFs play important metabolic effects. The secretion of alanine by CAFs supports malignant cell growth and may also have a positive effect on T cell function (21, 22).

## TIME suppresses intrinsic immunity

**Figure 1:**
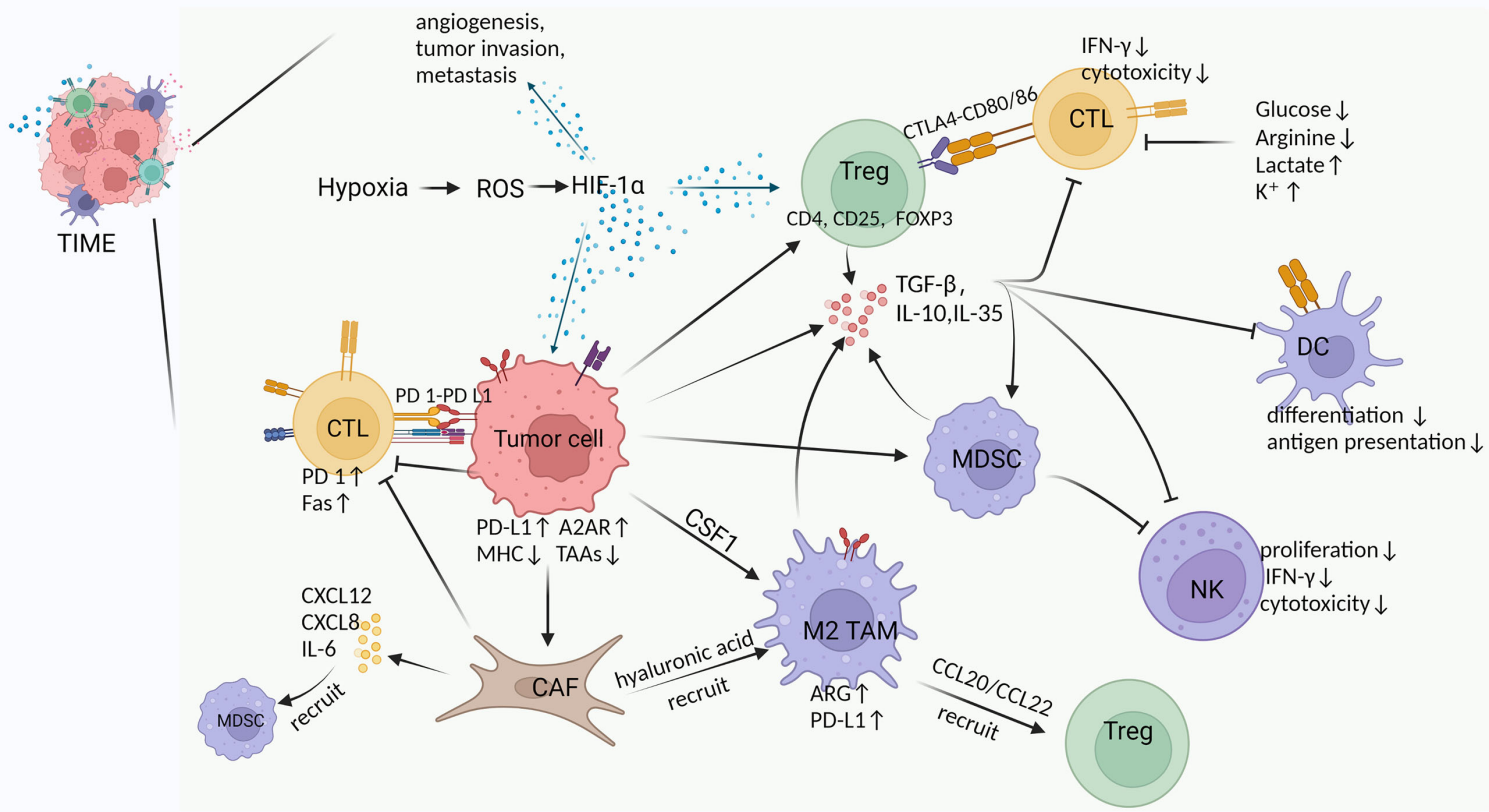
Cross-talk between various components in TIME. Hypoxia is an important feature of TIME in solid tumors, which exerts multiple effects by inducing HIF-1α, such as 1) promoting angiogenesis, tumor invasion and metastasis, 2) increasing Tregs abundance to suppress host immune response, and 3) upregulating PD-L1 and A2AR on tumor cells to evade immunity (26–30). Tumor cells induce and chemotactic immunosuppressive cells (Tregs, M2 TAMs, MDSCs, CAFs) to infiltrate in TIME by secreting TGF-β and CSF (31, 32). Moreover, tumor cells inherently upregulate PD-L1 and A2AR expression to suppress the immune function of CTLs, as well as downregulate MHC and tumor-associated antigens (TAAs) expression to reduce antigen presentation and immune activation (29, 30). Tregs produce multiple immunosuppressive cytokines and express CTLA4 to inhibit CTLs and DCs by binding CD80/86 (33). M2 TAMs in TIME suppress T cell function by upregulating ARG and PD-L1 expression while recruiting Tregs *via* CCL20/CCL22 (34–36). MDSCs produce inhibitory cytokines and inhibit the formation and cytotoxicity of NK cells by reducing NKG2D expression and IFN-γ secretion (37). CAFs secrete a large number of cytokines, chemokines and ECM, which exhibit immunosuppressive and tumor-supportive effects (31, 38, 39). For example, IL-6 from CAFs recruits MDSCs, CAFs reduce M1 macrophages while recruiting M2 TAMs *via* hyaluronic acid, upregulated expression of Fas and PD-1 suppresses and depletes T cells (40–42). Suppressive cytokines (e.g., TGF-β, IL-10, IL-35) can be produced by tumor cells and a variety of immunosuppressive cells in TIME to inhibit immune killing of CTLs, suppress the differentiation and antigen presentation of DCs, and restrain the proliferation and cytotoxicity of NK cells (43–46). In addition, glucose and arginine deficiency in TIME, as well as high lactate levels inhibit T cell function (47–51).

Although the immune system can clear tumors through the cancer-immune cycle, tumors often evade the body’s immune surveillance by shaping an inhibitory TIME. The complex interactions between the mediators of pro- and anti-tumor in TIME ultimately determine trends of anti-tumor immunity (23, 24). Among these, pro-tumor immune cells include regulatory T cells (Tregs), myeloid-derived suppressor cells (MDSCs), TAMs, CAFs, and tumor-associated neutrophils (TANs). In TIME, CAFs, TAMs and Tregs form an immune barrier to CTLs-mediated anti-tumor immune responses (15). In addition, pro-tumor immune cells and immunosuppressive factors (e.g., transforming growth factor β, interleukin-10) act synergistically to exert important immunosuppressive effects, including inhibition of differentiation and maturation of DCs, inhibition of NK cell toxicity, inhibition of antigen presentation, inactivation of the pro-apoptotic pathway, and disturbance of T cell receptor signaling (25) (**Figure 1**).

### Immunosuppression of tumor cells

Cytokines, chemokines and metabolites from tumor cells have a significant impact on TIME, such as transforming growth factor-β (TGF-β) and interleukin (IL)-10. Tumor cells inhibit the function of NK cells, CD8^+^ T cells and evade recognition and attack by the immune system. Most tumor cells express a large amount of stem cell factor, which induces mast cells to infiltrate the tumor site. Mast cells inactivate T cells and NK cells, as well as inhibit their anti-tumor activity (31). Colony stimulating factor 1 (CSF1) produced by tumor cells promotes differentiation of TAMs and production of granulocyte-specific chemokines in CAFs (31). CSF1 receptor inhibitor and CXCR2 antagonist treatment inhibit the recruitment of MDSCs to TIME, exhibiting significant anti-tumor effects (32). TIME also modify certain inflammatory cell types so that they present a pro-tumor phenotype, in particular many chronic inflammation-associated inflammatory cells promoting tumor progression (52, 53). Hypoxia is a prevalent feature in solid tumor TIME and contributes to the suppression of immune killer cells and protection of tumor cells from immune attack (26, 27). Hypoxia-induced factor 1α (HIF-1α) is a key regulator of adaptive responses to hypoxia, involved in angiogenesis, tumor invasion and metastasis, and increases Tregs abundance by inducing FOXP3 (28). Moreover, HIF-1α also increases PD-L1 expression on tumor cells and suppresses immune cell responses by targeting PD-1 on activated T cells. Tumor cells reduce the expression of MHC or tumor antigens to avoid recognition and clearance by immune cells (29). Adenosine A2a receptor (A2AR) expressed on tumor cells inhibits the activation of immune cells and its expression is associated with cytokines such as HIF-1α. A2AR blockade reduces CD4^+^ FOXP3^+^ Tregs infiltration and enhances the anti-tumor response of CD8^+^ T cells by attenuating hypoxic HIF-1α signaling (30).

### Immunosuppression of Regulatory T cells

Tregs are an immunosuppressive subset of CD4^+^ T cells characteristically expressing CD4, CD25, and FOXP3, and exhibit diversity and functional heterogeneity across tumor types. Hypoxia in TIME increases Tregs abundance by upregulating FOXP3 (28). Tregs, the important Tumor-Promoting Immune Cells, affect a variety of tumor-infiltrating immune cells by producing multiple immunosuppressive cytokines (such as TGF-β, IL-10 and IL-35) and exhibit a significant anti-tumor immune response (43). Tregs inhibit tumor killing by CTLs through TGF-β-dependent cell contact (54), inhibit the production of memory CTLs *via* cytotoxic-T-lymphocyte-associated protein-4 (CTLA-4) (33), and induction of CTLs death *via* granzyme B and perforin-dependent manner (55). In addition, Tregs inhibit IFN-γ secretion by CD8^+^ T Cells and promote the polarization of M2 TAMs (suppressing immunity) (56). Tregs-expressed CTLA-4 binds to CD80/CD86 on DCs to downregulate co-stimulatory signaling and inhibit DCs function (57). Tregs restrain NK cell proliferation, IFN-γ production, degranulation and cytotoxicity (58). Tregs-produced TGF-β and IL-35 enhance the function of MDSCs (59).

### Immunosuppression of tumor-associated macrophages

Macrophages account for >50% of tumor-infiltrating immune cells. According to function and cytokine secretion, macrophages are classified as classical activation (M1) with immunostimulatory function and alternative activation (M2) with immunosuppressive and tumor-supportive effects (60). Macrophages are referred to as TAMs in solid tumors, mainly M2, and there is a strong negative correlation between their presence and survival in a variety of solid tumors including breast, colon, bladder and lung cancers (61–64). TAMs are functionally heterogeneous and display remarkable plasticity, which allows macrophages to ‘switching’ into an ‘M2’ phenotype in TIME, associated with immunosuppressive, tumor angiogenic and metastatic consequences (44). In contrast to classical M1 macrophages, these M2 TAMs secrete large amounts of IL-10, and TGF-β, which exert anti-inflammatory effects (44). Hypoxia in TIME also increases arginase 1 (ARG1), VEGF, and macrophage-derived protein kinase signaling by activating mitogen-activated protein kinase signaling in TAMs (34, 65). ARG1 expression is upregulated in TAMs and tumor cells, inhibiting T cell activation by reducing arginine entry into tumor-infiltrating immune cells (34). M2 macrophages-derived CCL20/CCL22 is involved in the recruitment of Tregs (35). M2 TAMs also increases PD-L1 expression to attenuate the effect of CTLs (36).

### Immunosuppression by myeloid-derived suppressor cells

MDSCs represent a heterogeneous population of immature myeloid cells with different transcriptional activity and differentiation states, including granulocytic or polymorphonuclear MDSCs (PMN-MDSCs) and monocytic MDSCs (M-MDSCs) (66). They prevent T cell-mediated adaptive immune responses and killing of tumor cells *via* the innate immune system mediated by NK cells or TAMs (67). Among them, PMN-MDSCs produce ROS and reduce T-cell responses to antigens (66). M-MDSCs produce nitric oxide or differentiate into immunosuppressive macrophages to suppress immune activation (68, 69). Similar to Tregs, the secretion of IL-10 and TGF-β by MDSCs impairs CTLs function and facilitates the induction of Tregs formation (45, 46). MDSCs also cause arginine deficiency by consuming nutrients in the TIME, which in turn causes Teff cell inactivation (46). In a xenograft mouse model, MDSCs inhibit NK cells formation and cytotoxicity by reducing natural killer group 2 member D (NKG2D) expression and IFN-γ secretion (37).

### Immunosuppression of cancer-associated fibroblasts

CAFs generally exhibit immunosuppressive and tumor-supportive functions (70–72). CAFs secrete a large number of immunosuppressive cytokines and chemokines, such as CXCL12, CXCL8, IL-6, TNF, TGF-β, etc. (31). Among these, high levels of IL-6 recruit MDSCs, upregulate PD-L1 expression and induce tumor immunosuppression (40). CAFs inhibit the production of regulatory factors such as IFN-γ and TNF-α by T cells and block the migratory capacity of T cells (73). In addition, factors secreted by CAFs also reduce the migration of M1 macrophages and inhibit the pro-inflammatory function of M1 macrophages (41). Meanwhile, CAFs upregulate Fas and PD-1 expression on T cells and deplete CD8^+^ T cells by binding PD-L2 and FasL (42). CAFs remodel the ECM and protect tumor cells from CTLs, for example, hyaluronic acid produced by CAFs recruits TAMs to the TIME (38, 74, 75). In short, CAF-derived cytokines/chemokines mediate immune escape, growth and metastasis of tumors (39). The SynCon FAP DNA vaccine reduces the number of FAP^+^ CAFs by targeting Fibroblast activation protein (FAP), a major marker of CAFs, thereby inducing T cell activation and suppressing tumor metastasis (76, 77).

### Immunosuppression of TGF-β

In preinvasive disease, TGF-β mainly acts as a tumor suppressor. Once the tumor has invaded, TGF-β promotes tumor progression through epithelial mesenchymal transition, angiogenesis, tumor metastasis, proliferation and immunosuppression of CAFs in TIME (16, 78–81). RT-mediated reactive oxygen species(ROS) production can activate TGF-β (82). TGF-β promotes immunosuppressive TIME through its effects on all immune subgroups (16). For example, TGF-β promotes stromal fibrosis and immune escape, which exclude T cells from infiltrating into tumor tissue, thereby mediating resistance to T cell-directed immunotherapy (64).

### Immunosuppression of nutrient competition, metabolite and ion pooling

The high consumption of glucose and amino acids by tumor cells contributes to the achievement of tumor growth, metastasis and immune tolerance (83). Glucose deficiency leads to a decrease in glycolysis in immune cells, which hinders IFN-γ production and the function of CTLs (47). Arginine is exhausted by MDSCs and macrophages, resulting in arginine deficiency in TIME. The anti-tumor activity of T cells is inhibited due to protein biosynthesis-mediated cellular exhaustion (48, 49). Indoleamine 2,3-dioxygenase (IDO) is an important rate-limiting enzyme expressed in CAFs, macrophages, and tumor cells that catalyzes the production of kynurenine from tryptophan (84, 85). Tryptophan metabolites/enzymes suppress inflammatory responses by recruiting Tregs and inhibiting Teff cells proliferation (86, 87). High levels of extracellular lactate inhibited the proliferation and cytokine production of human CTLs (88). Excess lactate led to an acidic environment that reduced arginine concentrations in TIME by inducing ARG1 expression in macrophages, which in turn inhibited CD8^+^ T cell proliferation and function (50, 51). Intracellular lactate, a product of glycolysis, inhibits T-cell glycolysis by suppressing the mTORC1-mediated signaling pathway (89). The increase in extracellular fluid potassium ions caused by tumor necrosis leads to severe suppression of T cell effector function (90).

### Immunosuppression of blood vessels

The immunosuppressive properties of TIME promote vascular destruction, which limits the infiltration of cytotoxic T lymphocytes into the tumor and exacerbates hypoxia (91). And there is a functional defect in the emerging vascular network in TIME that promotes hypoxia formation (92).

In summary, according to the immune characteristics in TIME, Tumor immunophenotype is usually classified as “cold” or “hot” tumors, which suggests individualized clinical treatment options. In “hot” or “inflamed” tumors, high expression of PD-L1, enrichment of Th1-type chemokines, and a large number of NK cells, CD8^+^ T cells and APCs are found (93, 94). And it has been established that immune “hot” as a protective factor leads to better clinical outcomes when treated with anti-PD-1/PD-L1 (95). In contrast, “cold” tumors so-called “immune-desert” tumors, are characterized by a high number of Tregs and MDSCs, few NK cells, CD8^+^ T cells, Th1 cells and DCs, but abundant immunosuppressive cytokines (93, 94).

## Effects of RT on the TIME

RT is a form of local ablative physiotherapy, the principle of which was using high-energy radiation to treat localized tumors (96). In addition to damaging tumor cells through different pathways, RT also affects other components of the TIME, including immune cells, CAFs, etc. Besides, RT has both “Non-targeted” and abscopal effects on tumor cells. “Non-targeted” effects, also called bystander effects, are molecular signals from irradiated cells that affect adjacent non-irradiated tissues (97). An abscopal effect, explained by the regression of the tumor occurring at a site far from the radiation, is thought to be the result of a systemic immune response (98). Weichselbaum and colleagues experimentally confirmed that the host immune response was the primary cause of the RT response and not the intrinsic radio-sensitivity of the tumor cells (99). RT is involved in every process of the immune response, the recruitment and accumulation of T cells in tumors, the release and presentation of antigens, the initiation and activation of T lymphocytes, and the recognition and killing of tumor cells by T lymphocytes. The effect of RT on irradiated TIME is immunostimulatory or immunosuppressive, which is primarily influenced by the immune landscape of the tumor as well as the dose and fractionation of RT (100).

### RT and tumor cells

RT achieves single- and double-stranded DNA damage, mis-repair and chromosomal aberrations through the induction of ROS and reactive nitrogen species (RNS) (92, 101). When RT-induced damage is limited, cells initiate damage repair mechanisms (including DNA damage response, the unfolded protein response and autophagy) to ensure the survival of irradiated cells and re-entry into the cell cycle (102). However, when damage cannot be resolved by repair mechanisms, the molecular mechanism of adaptation to stress switches from a cytoprotective to a cytostatic or cytotoxic mode, usually in one of these forms, ultimately leading to cellular senescence or regulated cell death (RCD) (103). Moreover, both protective repair mechanisms and senescence or RCD have an impact on the local microenvironment and organismal homeostasis, not only through the production of many different cytokines and chemokines, but also through the involvement of damage-associated molecular patterns (DAMPs), ions, and metabolites (103). RT-driven DNA damage response (DDR) can mediate immunostimulatory effects (104). For example, irradiated cancer cells express NK cell-activating ligands (NKALs) on the cell surface after DDR, which support antigen-independent NK cell activation by binding to specific receptors on NK cells (105). NF-κB is sensitive to intracellular alterations that occur after RT, including DNA damage and oxidative stress (102). RT-induced initiation of NF-κB signaling increased the release of cytokines including TNF and IL-1β (106). Tumor cells and CAFs have a proficient autophagic response and successful autophagic response to RT not only preserves cellular viability but also facilitates the maintenance of immunosuppression by TIME (102). However, apoptotic RCD resulting from failing DDR, UPR and autophagic responses transmits danger signals to TIME *via* membrane exposure and secreted factors in response to lethally irradiation (102). In addition, radiation also induces a variety of non-apoptotic cell death signals, for example, RT-driven mitotic catastrophe activates cGMP-AMP (cGAMP) synthase (cGAS)-stimulator of interferon genes (STING) signaling *via* TBK1 and IRF3, thereby facilitating the secretion of large amounts of type I IFN (107, 108). Necroptosis is a major pro-inflammatory RCD modality that may ultimately lead to increased tumor infiltration of myeloid cells and CTLs (103, 109). Genetic data suggested that necroptosis was the predominant RCD mechanism in non-small cell lung cancer (NSCLC) cells expressing high RIPK3 levels after ablative hypo-fractionated RT (110). In contrast, a study by Sandy Adjemian et al. showed that necroptosis was not the predominant form of IR-induced death (111). The intrinsic radio-sensitivity of malignant cells exhibits intra- and inter-cancer variability, which depends not only on intrinsic cell characteristics (including efficient DDR, UPR and autophagic competence) but also on TIME factors (e.g. partial oxygen tension) (112–114). Depending on the radiation dose, high doses tend to trigger powerful cytotoxic effects and a strong immune response, while low doses tend to induce cellular senescence and acquire a senescence-associated secretory phenotype (SASP) mainly promoting immunosuppression (102, 115). Data suggest that when cells are irradiated with doses below 10 Gy, most DNA breaks can be repaired and the cells can resume their cell cycle, divide and remain viable. However, at doses higher than 10 Gy some DNA damage fail to repair, at which point mitotic catastrophe and many different forms of death occur (111).

Abscopal radiation-induced antitumor immune responses are rarely observed in clinical practice (116). Apparently, RT-induced antitumor immunity is dependent on RT-generated immune activation signals and immunosuppressive factors (4). One immunosuppressive component is TGF-β1, which promotes tumor progression, invasion and metastasis. Active TGF-β1 is produced in tumors after RT, particularly in endothelial cells undergoing low-dose ionizing-radiation (82, 117). TGF-β1 induces a phenotype of infiltrating inflammatory cells with immunosuppressive effects, e.g. TANs N2 with a protumor phenotype, TAMs M2 (118, 119). RT induces the expression of immunosuppressive molecules, such as PD-L1, through local cytokine-mediated extrinsic effects or P53-mediated intrinsic mechanisms (120, 121).

### RT and lymphocytes

Various subtypes of T cells have different resistance to RT, and unlike Th cells and CTLs, Tregs cells are relatively radioresistant (122). B cells and their precursor cells are highly sensitive to radiation-induced DNA damage (123). However, focal radiation treatment of tumor sites at 12-18 Gy using a mouse model suggests that radiation alters B-cell activation, differentiation and clonogenicity, prompting B cells resistance to tumorigenesis (124). Irradiation induces B cells maturation and activation as well as increases the differentiation of tumor antigen-specific plasma cells (124). RT induces the expression of CD20, a common surface antigen on B cells, which is now used as a target for some therapeutic strategies, such as radio-immunotherapy (125).

### RT and macrophages

Monocytes, the source of macrophages and DCs, show a high sensitivity to RT and oxidative damages that can lead to single- and double-strand DNA breaks (126). However, both macrophages and dendritic cells upregulate DNA damage repair mechanisms and display a relatively normal DNA repair damage response, leading to an increase in their radio-resistance (126). In TIME, a low-dose RT (LDRT) of 2 Gy induced the differentiation of iNOS^+^M1 macrophages promoting a pro-immunogenic environment (127). In contrast, higher RT doses promoted tumor infiltration *via* pro-tumorigenic M2-TAMs polarization (128, 129). In addition, high-dose RT (>8 Gy) may promote anti-inflammatory activation of macrophages (130) and doses of 20 Gy activate the M2 TAMs with pathogenic properties *via* induction of the immunosuppressive molecules COX-2/PGE2 and NO (128, 131).

### RT and DCs

Higher doses of irradiation (20 Gy) affect the function of DCs, leading to a reduction in the efficiency of antigen presentation and a reduced ability to induce T lymphocyte proliferation (126, 132). According to some reports, irradiated DCs secrete increased amounts of pro-inflammatory cytokines (including IL-1β and IL-12) and decreased amounts of anti-inflammatory cytokines such as IL-10 (133, 134). Fractionated RT along with anti-CTLA4 produced abscopal effects caused in part by an increased number of Batf3 DCs, which were abolished in Batf3-/- mice, confirming the important role of Batf3 DCs in RT-induced anti-tumor immunity (135–137).

### RT and Natural Killer Cells

Mature NK cells have been reported to be relatively radioresistant, while their precursors are radiosensitive (138). It is generally accepted that the effect of RT on NK cells is influenced by the radiation dose, with low doses of RT activating NK cells and high doses leading to impaired NK function (139). Low doses of RT (0.075 Gy to 0.15 Gy) triggered increased expression of IFN-γ and TNF-α *in vitro*, and doses of 0.1 Gy to 0.2 Gy resulted in NK activation in an vivo rat model (139). RT induces an ATM-dependent DNA damage response in NK that promotes immune response and reduces exhaustion (140). Radiation increased the ability of NK cells to kill experimental cells such as MCA105 and K562cells (141, 142), and studies using human primary NK cells or the NK-92 cell line also confirmed increased NK cell-mediated cytotoxicity after radiation (143). In addition, RT also induces the migration of NK cells towards the tumor with the help of the chemokine CXCL16/CXCR6 (144). Clinical data from patients with cervical cancer showed increased cytotoxic activity of circulating NK cells post-RT, suggesting systemic activation of NK cells (145). Some studies have also shown a decrease in circulating NK cells but an increase in robust TIM3^+^ NK cells after ablative RT (146). Conversely, several clinical studies have shown a decrease in NK cell activity post-RT (147–149).

### RT and CAFs

CAFs are extremely radio-resistant, do not trigger apoptosis even at high doses of radiation (e.g. 30 Gy) and maintain a strong immunosuppressive effect on activated T cells (in a single dose of 18 Gy) (73). However, post-RT CAFs become senescent and produce a different combination of immunomodulatory molecules (73). In particular, senescent CAFs secrete high levels of TGF-β1, which mediates T-cell rejection and facilitates the establishment of immunosuppressive TIME (150, 151). RT was associated with increased radio-resistance of tumor cells, including in NSCLC, which may be due to the pro-tumor activity of CAFs (152). The pro-tumorigenic nature of radiated CAFs is achieved by direct tumor cell stimulation and suppression of immune cells, including macrophages, DCs, NK cells and T cells (41, 70, 73). Differently, *in vivo* models have shown that irradiation of CAF (iCAF) alters pro-cancer characteristics and reduces tumor engraftment and angiogenesis (153). In conclusion, CAFs are the main drivers of becoming established immunosuppressive TIME post-RT.

### RT and vasculature, endothelial cells

There is evidence that single radiation doses of 5-10 Gy result in relatively mild vascular changes, while higher doses(>10 Gy) result in significant vascular damage, at which point reduced vascular flow due to endothelial cell death leads to hypoxia, reduced effector T cell recruitment and suppression of local immune responses in TIME (154). In addition, high doses of RT (HDRT) also exhibited pro-tumor effects by inducing HIF-1α/TGF-β signaling, increasing the number of CAFs and promoting fibrosis and remodeling of TIME (94).

### RT and chemokines, cytokines, and other soluble factors

Increased expression of type I IFNs post-RT stimulates the expression of chemokines CXCL9 and CXCL10, which recruit CXCR3-expressing T cells to the TIME (94). In addition, type I IFNs promote Battf3-expressing DCs to present antigens to CD8^+^ T cells and initiate anti-tumor immunity (155). IFN-γ promotes Th1 cells polarization and CTLs activation, but also upregulates PD-L1 expression in TIME (156, 157). HDRT induces the production of tumorigenic cytokines, such as HIF-1α/VEGF-A, which promotes the release of a large number of cytokines including IL-1, IL-6, IL-10, and TGF-β (94, 152). Among these, TGF-β exerts multiple immunosuppressive effects. TGF-β inhibits the expansion and cytotoxicity of CD8^+^ T cells, suppresses the differentiation of CD4^+^ T cells and induces Tregs transformation (94). In addition, RT-induced TGF-β signaling increases the number of CAFs whose release of CXCL12 binds to the ligands CXCR4 and CXCR7, exerting pleiotropic pro-tumor activity, including induction of tumor survival, metastasis and affecting immune cell infiltration, and function (158–162). RT-induced hypoxic TIME depletes glucose and essential amino acids, and metabolite accumulation occurs, such as lactate, adenosine, and kynurenine, which can blunt the function of CTLs while promoting the accumulation of Tregs (163).

Furthermore, while radiation initially induces an anti-tumor response, radiation also induces chronic inflammation and rebound immune suppression (64). During this phase, tumor-promoting macrophages are recruited to the tumor in a radiation dose-dependent manner, resulting in an immunosuppressive TIME that supports tumor regeneration or resistance. RT also induces HIF-1α which induces PD-L1 expression in tumor cells and TAMs, leading to resistance to RT and immunosuppression (164, 165). In addition, the inflammatory response induced by RT also induced upregulation of IDO, which increased TAMs and MDSCs in TIME, associated with tumor immunosuppression (166, 167). From this, it appears that radiation may have a temporary effect on the immune response to TIME, where there appears to be a window of anti-tumor response. The clinical data from the PACIFIC trial suggested that patients who started checkpoint suppression within 14 days of completing RT appeared to have better outcomes than those who started later (168).

## Reversal of RT: From immunosuppression to immunostimulation

A growing number of studies have confirmed that radiation increases the amount of MHC on the cell surface, leading to the expression and release of immunostimulatory cytokines and danger signals, which in turn leads to the activation of innate and adaptive immune responses (92, 169). (Variations of multiple factors in TIME post-RT are summarized in **Table 1**.) RT acts in several aspects of the immune response, transforming the immunosuppressed state into an immune activated state, e.g. increased infiltration of immune cells in TIME, activation of innate and adaptive immunity, enhanced existing T cell responses, neoantigen-induced immune responses and diminished immunosuppression.

**Table 1:** Summary of alterations in immunomodulatory factors post-RT.

Factors		Immunomodulation	Effect of RT	Ref.
**Immunocytes**	CD8^+^ T cells	Immunostimulation (tumor-specific cytotoxicity *via* MHC-I)	Increased infiltration and activation	(64, 135, 170–178)
	CD4^+^ T cells	Immunostimulation (Enhancing CTLs responses or exerting cytolysis *via* binding MHC-II)	Increased infiltration and activation	(64, 179, 180)
	DCs	Immunostimulation (Uptake of TAAs, cross-presentation, and initiation of tumor-specific CTLs)	Increased infiltration and activation	(173, 177, 181–184)
	NK cells	Immunostimulation (Killing tumor cells directly without prior sensitization or MHC restriction)	Increased infiltration and cytotoxicity	(180, 185)
	Tregs	Immunosuppression (Inhibiting CTLs and NK cells, enhancing MDSCs and M2 TAMs)	Decreased infiltration	(173–177)
	M1 macrophages	Immunostimulation (Production of pro-inflammatory cytokines)	Increased polarization	(127, 180, 181, 186)
	MDSCs	Immunosuppression (Secretion of immunosuppressive cytokines, inhibition of T cells and NK cells)	Decreased infiltration	(170, 173, 176, 187)
**Cytokines**	Type I IFNs	Immunostimulation (Recruitment of CD8^+^ T cells and CD4^+^ T cells, activation of DCs)	Increased expression	(94, 155, 188–190)
	TGF-β	Immunosuppression (Inhibiting CTLs and NK cells, inducing Tregs, M2 TAMs, and N2 TANs)	Decreased expression	(180, 186)
**Chemokines**	CXCL9, CXCL10	Immunostimulation (Recruiting CXCR3-expressing T cells)	Increased expression	(94)
	CXCL16	Immunostimulation (Recruiting CD8^+^ T cells)	Increased expression	(191)
	CXCL8	Immunostimulation (Inducing targeted migration of CD56dim NK cells)	Increased expression	(192)
**Adhesion molecules**	ICAM1, VCAM1	Immunostimulation (Recruitment and attachment of circulating leukocytes)	Increased expression	(92, 193, 194)
	E- selectin, P-selectin	Immunostimulation (Facilitating lymphocyte homing)	Increased expression	(195)
**DAMPs**	CRT	Immunostimulation (Prophagocytic signals for macrophages and DCs by binding to CD91 receptors)	Increased exposure	(196, 197)
	HMGB1	Immunostimulation (Activating T cells)	Increased release	(25, 174)
	ATP	Immunostimulation (Recruitment of monocytes and production of IL-1β)	Increased release	(198)
	Cytoplasmic DNA	Immunostimulation (Enhancing the expression of type I IFNs *via* cGAS-STING signaling)	Increased exposure	(188, 189, 199, 200)
**Cell surface molecules and receptors**	Fas	Immunostimulation (A specific death factor inducing apoptosis by binding to FasL)	Increased expression	(194, 201–203)
	MHC-I molecules	Immunostimulation (Transporting and displaying TAAs allowing CD8^+^ T cells to identify)	Increased expression	(201, 204)
	Hsp70	Immunostimulation (Activating monocytes, macrophages and DCs)	Increased exposure	(205)
	NKG2D	Immunostimulation (enhancing cytotoxicity of T cells)	Increased expression (CD4^+^ T cells)	(179, 206)
	NGK2D ligand	Immunostimulation (Sensitizing NK cell-mediated cytotoxicity)	Increased expression (tumor cells)	(207, 208)
	Neoantigen	Immunostimulation (Inducing neoantigen-specific CD8^+^ T cells and CD4^+^ T cells)	Increased expression	(203, 204, 209)
	CD47	Immunosuppression (An anti-phagocytic signal to promote immune evasion)	Decreased expression	(210)
	PD-L1	Immunosuppression (Inhibiting activation of T cells)	Increased expression	(120, 121, 176, 211)

### Increased immune cell infiltration in TIME

**Figure 2:**
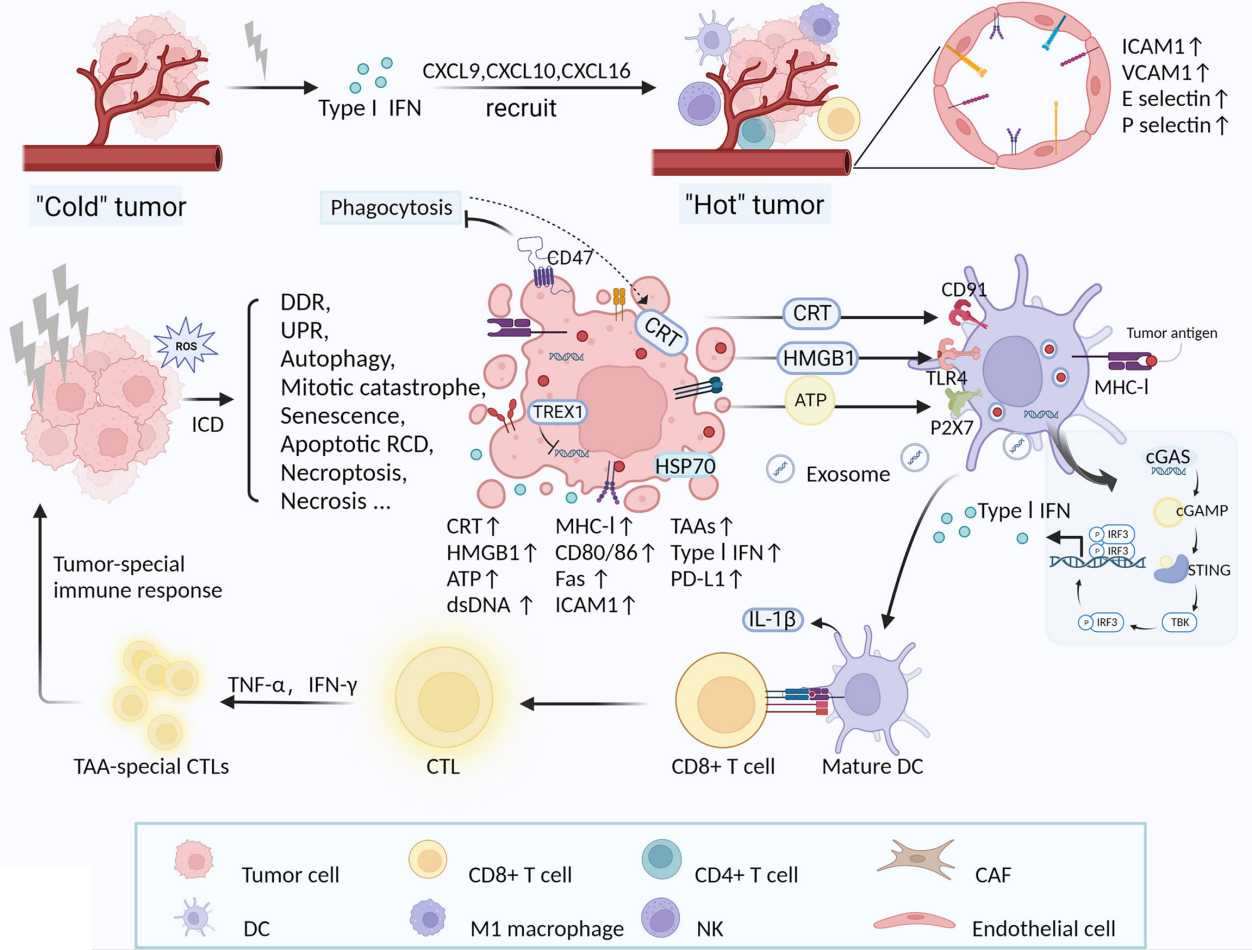
RT-induced increase in immune cells in TIME and tumor-special immune response activated by ICD. LDRT induces increased expression of a variety of molecules, including inflammatory cytokines (type I IFN), chemokines (CXCL9, CXCL10, CXCL16), adhesion molecules (ICAM1 and VCAM1) and E-/P-selectin, which facilitate the recruitment of multiple immune cells (DCs, NK cells, M1 macrophages, CD8^+^ and CD4^+^ T cells) (92, 94, 191, 193, 195). Concurrently, RT-induced vascular normalization also promotes immune cell infiltration in the TIME (181). Ultimately, RT transforms “cold” tumors (lymphocyte deficiency) into “hot” tumors sensitive to immunotherapy. RT directly or indirectly causes DNA damage in tumor cells and induces various forms of cellular responses and death, such as DDR, UPR, autophagy, mitotic catastrophe, senescence, apoptotic RCD, necroptosis, necrosis, etc (102, 103, 107, 108). Exposure and release of multiple DAMPs are critical for RT-induced ICD, including CRT (binding to CD91), HMGB1 (binding to TLR4), ATP (binding to P2Y2), and dsDNA (entering DCs) (25, 196–198, 214). DAMPs or danger signals recruit DCs and other APCs into irradiated TIME and to promote the maturation and activation of DCs. Mature DCs enhance uptake of tumor-associated antigens (TAAs) and subsequent cross-presentation with CD8^+^ CTLs, thereby initiating a tumor-specific adaptive immune response (181–183). During the process, dsDNA from irradiated cells activates the cGAS-STING pathway signaling *via* TBK1 and IRF3 in host DCs and tumor cells, culminating in the production of type I IFN (188, 189, 199, 200). Type I IFN in TIME is thought to be crucial for the induction of anti-tumor immune responses by RT (165, 190). In addition, RT enhances the immunogenicity of tumor cells by modulating the expression of cell surface molecules and receptors, and enhances the existing immune response, such as MHC-I, stimulatory molecules (CD80, CD86), adhesion molecules (ICAM1), death receptors (Fas) (204, 207, 215–218). PD-L1 expression is increased on tumor cells post-RT, which is one of the important targets for ICB therapy (120, 121, 164, 165, 176).

A single irradiation with 2 Gy increased the ability of tumor-specific CD4^+^ and CD8^+^ T cells to migrate into the tumor (127). LDRT-induced expression of inflammatory cytokines (IL-1β, TNF-α and type I and II IFN) and endothelial cell-activated adhesion molecules (ICAM1 and VCAM1) facilitates extravasation and activation of immune cells (92, 193). RT induced the expression of E- and P-selectin on the surface of vascular endothelial cells, facilitating lymphocyte homing (195). RT induced polarization of M1 macrophages and secretion of NO *via* iNOS activation, promoting normalization of blood vessels and facilitating adhesion and infiltration into the TIME (181). Cytoplasmic DNA produced by irradiated tumor cells is sensed by cGAS, which enhances the expression of type I IFNs through cGAS-STING signaling in host immune cells and tumor cells (188, 189). Increased expression of type I IFNs after RT stimulates the expression of chemokines CXCL9 and CXCL10, which recruit CXCR3-expressing T cells to the TIME (94). Similarly, RT induced CXCL16 to interact with CD8^+^ T cells to promote their recruitment activity (191). Tumor cells with senescent characteristics induce targeted migration of CD56dim NK cells by secreting CXCL8, which in turn initiates an innate anti-tumor immune response (192). In addition, radiation generates a pro-inflammatory microenvironment with remodeling of the vasculature, allowing T cells extravasation and tumor destruction (212). RT makes refractory “cold” tumors sensitive to immune checkpoint inhibitors by promoting the recruitment of anti-tumor T cells (213). One study showed that HDRT reshaped the immunosuppressive tumor microenvironment, leading to a significant increase in CD8^+^ T cell tumor infiltration, while suppressing MDSCs, however, the number of CD8^+^ T cells decreased when extended fractionated radiation was given (170). In studies on oral squamous cell carcinomas, metastatic renal cell carcinomas, and soft tissue sarcomas, neo-adjuvant RT increased the number of locally infiltrating immune cells in a variety of tumors, including CD4^+^, CD8^+^, and CD20^+^ TILs (64, 171, 172). Recent experiments in both mouse models and patient tumors have found that LDRT induces predominantly infiltration of CD4^+^ T cells with Th1 signatures in TIME (179). (**Figure 2**)

### Innate and adaptive immune response activation

A series of studies have demonstrated that radiation induces innate and adaptive immune response activation, of which RT-induced ICD is a very important mechanism that alters intracellular immunogenicity through external stimulation (182, 214, 219). ICD is characterized by the translocation of the calreticulin (CRT), the release of high-mobility group box 1 (HMGB1) protein and the release of ATP following apoptosis (214). Among these, the ER-derived proteins CRT translocated from the ER to the cell surface are key to the ICD, which binds to CD91 receptors as prophagocytic signals for macrophages and DCs (196, 197). HMGB1 stimulates the TLR4/MyD88/TRIIF pathway and activates T cells (25). In TIME, these factors act synergistically as DAMPs or danger signals to recruit DCs and other APCs into irradiated TIME and to promote the maturation and activation of DCs. Mature DCs enhance uptake of tumor-associated antigens (TAAs) and subsequent cross-presentation with CD8^+^ CTLs, thereby initiating a tumor-specific adaptive immune response, activating T cells and forming memory phenotypes (181–183). The current study demonstrates that during RT-mediated ICD, tumor-derived dsDNA enters the cytoplasm of DCs and activates the cGAS-STING DNA-sensing pathway signaling a type-I IFN response in which TREX1 exerts an inhibitory effect by degrading DNA (199, 200). STING induces IFN-β transcription and type I IFN expression, which are required for DC activation, ultimately leading to cross-presentation of TAAs and initiation of tumor-specific CTLs (155, 190). In addition to host immune cells, DNA-sensing pathways in tumor cells were also activated, which increase type I IFN production (188, 189). Inflammatory pathways activated by STING ligands have adjuvant activity enhancing tumor-specific adaptive immune responses post-RT (220). ATP is involved in the recruitment of monocytes into tumors (via P2Y2 receptor) and in the production of IL-1β (via P2RX7 receptor and inflammasome NLRP3), which is required for the activation of T cells (198). Unlike Apoptotic cells, which are normally cleared *via* the anti-inflammatory pathway, necrotic cells are immunogenic due to loss of membrane integrity and sustained release of DAMPs, inducing strong immune and inflammatory responses (207, 221). For example, the apoptosis inhibitor zVAD-fmk effectively blocked programmed cell death and induced necrosis as a form of ICD, and its combination with radiation altered the infiltration of immune cells in TIME, i.e. increased DCs and CD8^+^ T cells and decreased Tregs and MDSCs (173).(**Figure 2**)

### Enhancement of existing T-cell responses

Preclinical studies have shown that SBRT can also enhance immunogenicity by modulating the expression of cell surface molecules and receptors to reinforce existing immune responses, such as MHC-I, stimulatory molecules (e.g. CD80, CD86), adhesion molecules (e.g. ICAM1), death receptors (e.g. Fas), NKG2D ligands, heat-shock proteins (e.g. HSP70), endoplasmic reticulum (ER)-derived calreticulin, etc. (204, 207, 215–218). Garnett et al. investigated the increase in five cell surface antigen proteins (including Fas, MHC-I, ICAM-1, CEA, or mucin-1) post-RT in 23 human carcinoma cell lines and the results suggested that 91% of human carcinoma cell lines showed dose-dependent increases in at least one antigen (201). Among other things, radiation increased Fas gene expression in tumor cells of CEA-expressing mice, thereby enhancing their sensitivity to CEA-specific CTL-mediated killing (201).

Several studies have confirmed that radiation increases the expression of tumor cell surface antigens, particularly MHC class I/II antigens, and that its expression upregulation is dose-dependent (222, 223). RT upregulates MHC-I molecules and generates unique MHC-I antigenic peptides that promote antigen-specific CTLs responses, one of the important mechanisms of RT induced immune sensitization (201, 204). RT has also been shown to enhance the susceptibility of tumor cells to immune-mediated cytotoxicity *via* the Fas/FasL pathway, a key mechanism for cell death mediated by NK cells and CTLs (202). Chakraborty et al. demonstrated that RT can upregulate Fas and ICAM-1 expression on MC38 mouse colon cancer cell lines in a dose-dependent manner (194). Hsp70 translocates from the cytoplasm to the extracellular matrix by binding to CD14, CD40, CD91, Lox1 and Toll-like receptors to activate monocytes, macrophages and DCs (205).

NKG2D is an essential costimulatory receptor expressed mainly on CD8^+^ T cells and NK cells, contributing to enhanced cytotoxicity of T cells and prevention of Fas-mediated autophagy (224–226). In addition, NKG2D^+^ CD4^+^ T cells were found in cervical carcinoma while missing on CD4^+^ T cells in the physiological state (179, 206). Tumors evade NKG2D through multiple mechanisms and soluble NKG2D ligands improve ICB effects, suggesting an significant anti-tumor function of the NKG2D pathway (227–230). RT was also found to upregulate NGK2D ligand expression on tumor cells, making them more sensitive to NK cell-mediated cytotoxicity (207, 208). Recently Fernanda G Herrera and colleagues found an increase of NKG2D^+^ CD4^+^ T cells in TIME post-LDRT and exhibited proliferative capacity (179). Furthermore, an elevated expression of NKG2D ligand RAE1 was observed in DCs, supporting a functional cross-talk between DCs and CD4^+^ T cells *via* NGK2D pathway (179).

### Neoantigen-induced immune responses

Silvia C Formenti et al. reported that RT in combination with CTLA-4 blockade induced anti-tumor responses in chemotherapy-refractory metastatic non-small cell lung cancer (NSCLC), where TCR-Seq analysis of responding patients suggested that CD8^+^ T cells expanded rapidly *in vivo* due to the recognition of a new antigen encoded by a gene that was upregulated by radiation (209). RT upregulated the expression of genes containing immunogenic mutations in a mouse model of triple-negative breast cancer with poor immunogenicity and increased tumor cell surface death receptors Fas and DR5, with the result that neoantigen-specific CD8^+^ T cells and CD4^+^ T cells preferentially killed irradiated tumor cells as well as promoted epitope spreading (203). This suggests that exposure to RT-induced immunogenic mutations stimulates a systemic anti-tumor response. Factors released from dead cells may be the source of radiation-associated antigenic proteins (RAAPs) (231). RT is able to modulate the peptide repertoire of irradiated cells, and in particular, radiation induces the expression of novel proteins that result in unique MHC-I antigenic peptides that enhance polyclonal antigen-specific CTLs responses (204).Whole-exome sequencing of NSCLC treated with PD-1 blockade confirmed treatment response was better when there was increased mutational burden, higher neo-antigenic burden and mutations in the DNA repair pathway (232). This suggests that the response to immunotherapy after RT is associated with irradiation-induced neoantigens.

### Decreased immunosuppression

Radiation also reduces the immunosuppressive properties to achieve remodeling of the TIME. TNF production by radiation-activated T cells leads to direct elimination of MDSCs locally and in the system (187). In contrast, found in experiments of RT combined with DNA vaccines, RT induced a decrease in Tregs, but not MDSCs (174). The transmembrane protein CD47 is overexpressed in most cancer cells and acts as an anti-phagocytic signal to promote immune evasion, with downregulation of expression in the presence of radiation exposure (210).

### The effect of RT-induced reconfiguration of the TIME on the effectiveness of immunotherapy sensitization

Immunotherapies designed to activate the patient’s immune system to kill cancer cells include chimeric antigen receptor T-cell therapy (CAR-T), immune-checkpoint blockade (ICB), and tumor vaccines. ICB is the most commonly used immunotherapy option. Immune checkpoints are a series of inhibitory pathways present in the immune system that are essential for the maintenance of self-tolerance and facilitate the regulation of duration and amplitude of physiological immune responses in order to mitigate additional tissue damage. Unfortunately, tumors use certain immune checkpoint pathways as the primary mechanism of immune resistance (211). CTLA-4 binds to its ligands B7-1 (CD80) and B7-2 (CD86) to generate inhibitory signals that suppress T cell activation and cytokine production and protect tumor cells from T cell attack (233). PD-1, mainly expressed in activated T cells, B cells and macrophages, binds to ligands (PD-L1 and PD-L2) to inhibit T cell activity, induce apoptosis of tumor-specific T cells and suppress Tregs apoptosis (234). PD-L1 is expressed on tumor cells, immune cells and epithelial cells, whereas PD-L2 is only induced on antigen-presenting cells (235). PD-L1 is overexpressed on tumor cells and is thought to be associated with immune escape. ICB alleviate the functional suppression of T cells and have been used to shift the balance of TIME from an immunosuppressed to an immune activated state, resulting in a sustained and durable anti-tumor response at multiple lesion sites (236). Anti-PD-1, anti-PD-L1 and anti-CTLA-4 are currently FDA-approved treatment options for a variety of cancer types (237). In addition, some emerging immune target studies have recently emerged, for example, lymphocyte activation gene 3 (LAG-3) overexpression in Tregs produces the immunosuppressive cytokines IL-10 and TGF-β, which inhibit the activity of effector T cells, and whose expression levels correlate with tumor progression and poor prognosis (238). Dual blockade of LAG-3 and PD-1 also increased the number of tumor-infiltrating CD8^+^ T cells and reduced Tregs, thereby synergistically enhancing anti-tumor immunity (239). Dual blockade of PI3k-γ and CSF-1R promotes a shift in polarization state from M2 TAMs to M1 macrophages, reduces infiltration of MDSCs and enhances CD8^+^ T cell activation in TIME (240). DC-based vaccines can activate T-cell responses by removing inhibition of antigen presentation (241).

As radiotherapy can produce anti-tumor immune response and a control mechanism of suppressive tumor immune response, thus the combination of RT and drugs targeting tumor immunosuppression enhances the anti-tumor immune response and improves the efficacy of single modality therapy (64). Currently, numerous preclinical and clinical studies reveal the synergistic effect of RT with ICB (155, 165, 179, 180, 209, 242–245), and part of the relevant clinical trials are summarized in **Table 2**. Jing Zeng and colleagues showed that the combination of PD-1 blockade and local RT led to long-term survival in mice with *in situ* brain tumors compared to single radiation or immunotherapy, and that immunological data showed increased infiltration of CTLs(CD8^+^/IFN-γ^+^/TNF-α^+^) and reduced infiltration of Tregs (CD4^+^/FOXP3) in the combined treatment group (175). Single-cell RNA-sequencing revealed a significant increase in B cells germinal center formation after PD-1 blockade and radiotherapy (124). To take advantage of the enhanced radiation-induced endogenous anti-tumor immune response, increased PD-L1 expression on tumor cells or infiltrating immune cells must be counteracted by blocking the PD-1/PD-L1 pathway (211). In studies on NSCLC, PD-L1 expression was increased both *in vitro* and *in vivo* after conventionally fractionated radiation. Further studies showed that RT combined with anti-PD-L1 antibody enhanced anti-tumor immune responses by promoting CD8^+^ T cell infiltration and reducing MDSCs and Tregs cell aggregation (176). Xiaoqiang Qi et al.’s study of the therapeutic effect of Minimally invasive radiofrequency ablation (RFA) combined with sunitinib in an HCC model showed that the combined treatment increased the frequency of CD8^+^ T cells and DCs, reduced Tregs infiltration, and activated tumor-specific antigen (TSA) immune response, ultimately favoring inhibition of HCC growth (177). In addition, RFA caused the upregulation of PD-1 in tumor-infiltrating T cells by promoting hepatocyte growth factor (HGF) expression, which was inhibited by sunitinib treatment (177). The combination of anti-CTLA-4 antibody and fractionated RT regimens showed an enhanced antitumor response at the primary site *in situ* and an abscopal effect was observed (135). The frequency of CD8^+^ T cells producing tumor-specific IFN-γ correlates with secondary tumor suppression (135). Ming-Cheng Chang et al. demonstrated that local RT stimulated DCs by inducing apoptosis and HMGB-1 release. RT combined with DNA vaccine increased the number of antigen-specific CD8^+^ CTLs and enhanced antitumor efficacy and suggested that biweekly moderate radiation dose was a more optimal choice (174). Chemoradiotherapy-exposed TIMEs were highly enriched with newly infiltrated tumor-specific CD8^+^ T cells and tissue-resident memory T cells, moreover, the authors found that chemoradiotherapy combined with dual CTLA-4 and PD-1 blockade achieved optimal anti-tumor effects (254). As recent studies have shown, LDRT combined with ICB improved the anti-tumor outcome of ICB by supporting M1 macrophages polarization, enhancing NK cells infiltration and reducing TGF-β levels. Moreover, Depletion of CD4^+^ T cells and NK cells attenuated this anti-tumor effect, suggesting a key role of both cells in the anti-tumor immunity (180). Similarly, Preclinical and clinical studies supported LDRT induces predominantly infiltration of CD4^+^ T cells with Th1 signatures in TIME (179).

**Table 2:** Landmark clinical trials of RT combined with immunotherapy for the treatment of cancers.

First Author	Patients	Cancer types	RT planning	Immunotherapy planning	Treatment schedule	Outcomes	Data source
**Willemijn S M E Theelen** (246)	92	Advanced Non-Small Cell Lung Cancer	8Gy×3	Pembrolizumab 200 mg/kg q3w	Pembrolizumab alone vs. pembrolizumab + SBRT	ORR 18% vs. 36%; p = 0.07 mPFS 1.9 vs 6.6; p = 0.19 mOS 7.6 vs. 15.9; p = 0.16	https://clinicaltrials.gov/ct2/show/NCT02492568
**Willemijn S M E Theelen** (247)	148	Metastatic non-small-cell lung cancer	8Gy×3, or 12.5Gy×4, or 3Gy×15	Pembrolizumab 200 mg/kg q3w	Pembrolizumab alone vs. pembrolizumab + SBRT	Best ARR 19.7% vs 41.7% (OR 2.96, p=0·0039) Best ACR 43.4% vs 65.3% (OR 2.51, p=0·0071) mPFS 4.4months vs 9.0 months (HR 0.67, p=0.0071) mOS 8.7months vs 19.2months (HR 0.67, p=0.0004)	https://clinicaltrials.gov/show/NCT02492568 https://clinicaltrials.gov/show/NCT02444741
**Narek Shaverdian** (248)	97	Stage IV advanced Non-Small Cell Lung Cancer	Previously received any RT	Pembrolizumab 2 mg/kg q3w or 10 mg/kg q3w or 10mg/kg q2w	Pembrolizumab with a history of RT vs pembrolizumab alone	mPFS 4.4 vs. 2.1; p = 0.019 mOS 10.7 vs. 5.3; p = 0.026	https://clinicaltrials.gov/ct2/show/NCT01295827
**Yijun Hua** (249)	25	recurrent nasopharyngeal carcinoma	2.2Gy×30	Toripalimab 240mg q3w	Toripalimab + RT	79.2% overall response, 95.8% disease control	https://clinicaltrials.gov/ct2/show/NCT03854838
**Shankar Siva** (250)	30	oligometastatic clear cell renal cell carcinoma	18-20Gy×1	Pembrolizumab 200mg q3w	Pembrolizumab+ RT	1- and 2-yr OS 90% and 74%, 1- and 2-yr PFS 60% and 45%	https://clinicaltrials.gov/ct2/show/NCT02855203
**Alice Y Ho** (251)	17	metastatic triple-negative breast cancer	600 cGy×5	Pembrolizumab 200mg q3w	Pembrolizumab+ RT	ORR 17.6%, CR 17.6%	https://clinicaltrials.gov/ct2/show/NCT02730130
**Chad Tang** (252)	31	Metastatic liver or lung Cancer	12.5Gy×4 or 6Gy×10	Ipilimumab 3 mg/kg q3w	Ipilimumab+ RT	10% PR, 13% SD	https://clinicaltrials.gov/ct2/show/NCT02239900
**Hari Menon** (253)	26	Metastatic Malignant Solid Neoplasm	7.3Gy (1.1-19.4Gy)	Pembrolizumab 200 mg/kg q3w, or Ipilimumab 3 mg/kg q3w	38 low-dose lesions vs 45 no-dose lesions	PR/CR 58% vs 18% (P = 0.0001) median change for longest diameter size -38.5% vs 8% (P < 0.0001)	https://clinicaltrials.gov/ct2/show/NCT02239900 https://clinicaltrials.gov/ct2/show/NCT02444741 https://clinicaltrials.gov/ct2/show/NCT02710253

ORR, overall response rate; mPFS, Median progression-free survival; mOS, median overall survival; ARR, out-of-field (abscopal) response rate; ACR, abscopal disease control rate; CR, complete responses; PR, partial response; SD, stable disease.

It is currently believed that HDRT (>5 Gy per fraction) is of limited value in tumor immunomodulation due to the presence of inherent toxicity and immunosuppression, whereas more recent studies have elaborated that LDRT (<3Gy per fraction) stimulates innate and adaptive immune responses, as well as improves the sensitivity of primary and metastatic lesions to ICBs, which is expected to improve cancer treatment outcomes by combining ICB (7). Three recent preclinical studies (1 Gy in lung adenocarcinoma model, 2.5 Gy in melanoma tumors model, 0.5-2 Gy in ovarian cancer model) all elucidated that LDRT acts as a modifier of immune response, remodeling TIME, significantly increasing infiltration of effector immune cells including tumor-infiltrating myeloid cells, DCs, NK cells, CD4^+^ and CD8^+^ effector T cells, etc., and was superior to either treatment alone in combination with ICB (178–180). Currently, for “cold” tumors ICB is not effective (255, 256). The corresponding Phase I clinical studies(https://clinicaltrials.gov/ct2/show/NCT02710253, https://clinicaltrials.gov/ct2/show/NCT03728179) were conducted in patients with a variety of “immune desert” tumors, including but not limited to advanced melanoma, anaplastic thyroid carcinoma, and metastatic ovarian cancer, demonstrating the safety, feasibility, and significant therapeutic efficacy of RT in combination with ICB.

It is generally accepted that TGF-β signaling is a strong regulator of radiation response in normal and tumor tissues (257). A preclinical study showed that concurrent administration of TGF-β blockade and RT followed by a PD 1 inhibitor improved tumor control and prolonged survival in a mouse model of metastatic cancer (258). The combination of RT and TGF-β blockade thus offers a new direction for personalized cancer therapy. In recent years, multiple studies using radiosensitizer have revealed potent RT-induced antitumor immunity, while also providing new options for radio-immunotherapy. Experiments by Kaiyuan Ni et al. observed that intra-tumor injection of radiosensitizer repolarized M2 TAMs to M1 macrophages, reduced intra-tumor TGF-β and collagen density, as well as inactivated CAFs. When intravenous radiosensitizer was combined with ICB, the mouse model exhibited enhanced T cells infiltration and a robust abscopal effect (186). Radiosensitizer acting on the STING pathway significantly promoted the activation of DCs and enhanced systemic immune responses against primary and metastatic tumors (184). Recently developed biogenetic gold nanoparticles (Au@MC38), a radiosensitizer, intensified radiation-induced DNA damage and ROS production, exacerbated apoptosis and necrosis, enhanced ICD-mediated immune responses, and achieved a satisfactory survival benefit in combination with ICB (259). Recently, additional pathways have been identified that may be involved in the radio-immunotherapy process. For example, tumor-induced CD45^-^Ter119^+^CD71^+^ erythroid progenitor cells (Ter cells) promote tumor progression by secreting artemin (ARTN), a neurotrophic peptide. Both topical RT and anti-PD-L1 treatment reduced Ter cell abundance and ARTN secretion in mice by an IFN- and CD8^+^ T cell-dependent manner (260).

Regarding the fractionation and dose of RT, M Zahidunnabi Dewan et al. showed that fractionated but not single-dose RT induced local and systemic anti-tumor immune responses when in combination with anti-CTLA-4 antibody (135). Single radiation doses (>12 Gy) may attenuate immunogenicity through TREX1 induction, while hypo-fractionated regimens (i.e., 8 Gy × 3) may be more effective when used in combination with immunotherapy (188). Fractionated doses of 2.5 Gy×4 and 15 Gy×2 produced higher NK cytotoxicity than single doses (e.g. 30 Gy or 10 Gy) (185). Differently, a study by Byron C Burnette and colleagues suggested that local high single dose RT promotes type I IFN production, initiating a cascade of innate and adaptive immune attacks against tumors by enhancing the ability to prime trans-tumor infiltrating dendritic cells (TIDC) (189). Latest animal and clinical studies indicated that when tumor burden was high, it was necessary to combine high-dose RT, low-dose RT and ICB therapy to achieve optimal therapeutic effects, specifically, HDRT (12 Gy×3) to target primary tumors that had activated T cells, while LDRT (1 Gy×2) targeted metastatic lesions to modulate immunosuppressive stroma and sensitize ICB (180).Thus, fractions and doses can significantly alter the immune response to TIME radiation. Primarily, immune cells must be recruited into the tumor by RT and immune activation achieved, followed by additional immunotherapy in order to exert a stronger anti-tumor immune effect. However, more data are urgently needed to draw more consistent conclusions about RT activation of the immune response and the optimal dose and fractionation in combination therapies with immunotherapy. And there may not be a so-called optimal RT fraction and dose, but different fractions and doses may be the most effective way to utilize the immunogenic properties of radiation in multimodal tumor therapies (4). Regarding the timing of immunotherapy after RT, studies have shown that immune cells migrate into the TIME within two days after the last radiation and remain there for several days, suggesting that immunotherapy is best applied in the middle to end of the treatment cycle (96). Additional studies have also shown that the combination of anti-PD-1 Ab one week after the last irradiation did not improve the tumor effects of RT (165). Thus, hypo-fractionated RT may predominate and longer radiation pauses allow time for the immune system to activate and function (96).

## Conclusion

RT remodels the suppressive TIME and mobilizes immune response, which creates the conditions for immunotherapy to work better and thus act locally and systematically against tumors. RT in combination with additional immunotherapy is a promising approach to induce specific anti-tumor immune responses. Accumulating clinical and preclinical data suggest that the immunogenic effects of radiotherapy may convert “cold” tumor into “hot” lesion with massive immune cell infiltration, thereby sensitizing unresponsive tumors to immunotherapy (213). There is a very delicate balance between activation of the immune system and RT-induced immunosuppression, depending on the specific radiation timing, fractionation, and dosing regimen. There is a need to initiate clinical trials and preclinical studies aimed at systematically evaluating the effects of different grading and treatment regimens to gain more insight into the optimal dose and schedule that may be able to induce synergy between immunotherapy and RT. As different immune cell types, with different states of differentiation, exhibit different radio-sensitivities, the selection of the most suitable radiotherapy regimen for combination with immunotherapy must carefully consider the radio-sensitivity of TIME and circulating lymphocytes. In addition to this, which LDRT technology is preferable and which drug combinations benefit the most in radio-immunotherapy are critical issues to be explored more thoroughly in the future. Overall, although there is strong evidence from preclinical work that radiotherapy and immunotherapy are synergistic, clinical reports detailing the interaction of radiotherapy and immunotherapy are limited, and are currently under development.

## Author contributions

SZ, YW, and JT wrote and edited the manuscript. MC designed the study and modified grammatical errors. All authors contributed to the article and approved the submission.

## Conflict of interest

The authors declare that the research was conducted in the absence of any commercial or financial relationships that could be construed as a potential conflict of interest.

## Publisher’s note

All claims expressed in this article are solely those of the authors and do not necessarily represent those of their affiliated organizations, or those of the publisher, the editors and the reviewers. Any product that may be evaluated in this article, or claim that may be made by its manufacturer, is not guaranteed or endorsed by the publisher.
